# Secondary Hypertrophic Osteoarthropathy (HOA) or Pierre Marie-Bamberger Syndrome Revealing Lung Adenocarcinoma

**DOI:** 10.7759/cureus.96889

**Published:** 2025-11-15

**Authors:** Hafsa Jamal Eddine, Othmane Zouiten, Leila Afani, Mohamed El Fadli, Rhizlane Belbaraka

**Affiliations:** 1 Medical Oncology Department, Mohammed VI University Hospital, Marrakesh, MAR

**Keywords:** adenocarcinoma, hypertrophic osteoarthropathy, lung tumors, paraneoplasic syndrome, pierre marie-bamberger syndrome

## Abstract

Digital clubbing, excruciating polyarthritis, and periosteal thickening of long bones are all features of the uncommon clinical syndrome known as hypertrophic osteoarthropathy (HOA). The most frequent association is lung cancer, which often presents as a paraneoplastic syndrome. We report the case of a 54-year-old male smoker who presented with joint pain and digital clubbing. Further investigations revealed a stage IV primary lung adenocarcinoma. Bone scintigraphy, performed after the diagnosis of the primary tumor, demonstrated increased uptake in the cortical bone of the lower limbs, with higher activity in both the axial and peripheral skeleton. The diagnosis of secondary HOA was established. The patient received palliative chemotherapy, which resulted in partial improvement of the articular symptoms. This case highlights the importance of considering HOA as a possible paraneoplastic manifestation, particularly in patients with lung cancer.

## Introduction

Hypertrophic osteoarthropathy (HOA) was first described in 1891 by Pierre Marie and Eugen von Bamberger. It is a rare condition characterized by the classic triad of digital clubbing, polyarthritis, and periostitis of the long bones [1-2]. HOA can be classified into two forms: primary HOA, or pachydermoperiostosis, a rare autosomal dominant familial disorder accounting for 3-5% of cases, and secondary HOA, also known as Pierre Marie-Bamberger (PMB) syndrome, which most commonly occurs in association with malignant pulmonary diseases, particularly non-small cell lung carcinoma [3].

We report the case of a 54-year-old male smoker who presented with PMB syndrome and was subsequently diagnosed with stage IV primary lung adenocarcinoma.

## Case presentation

**Figure 1:**
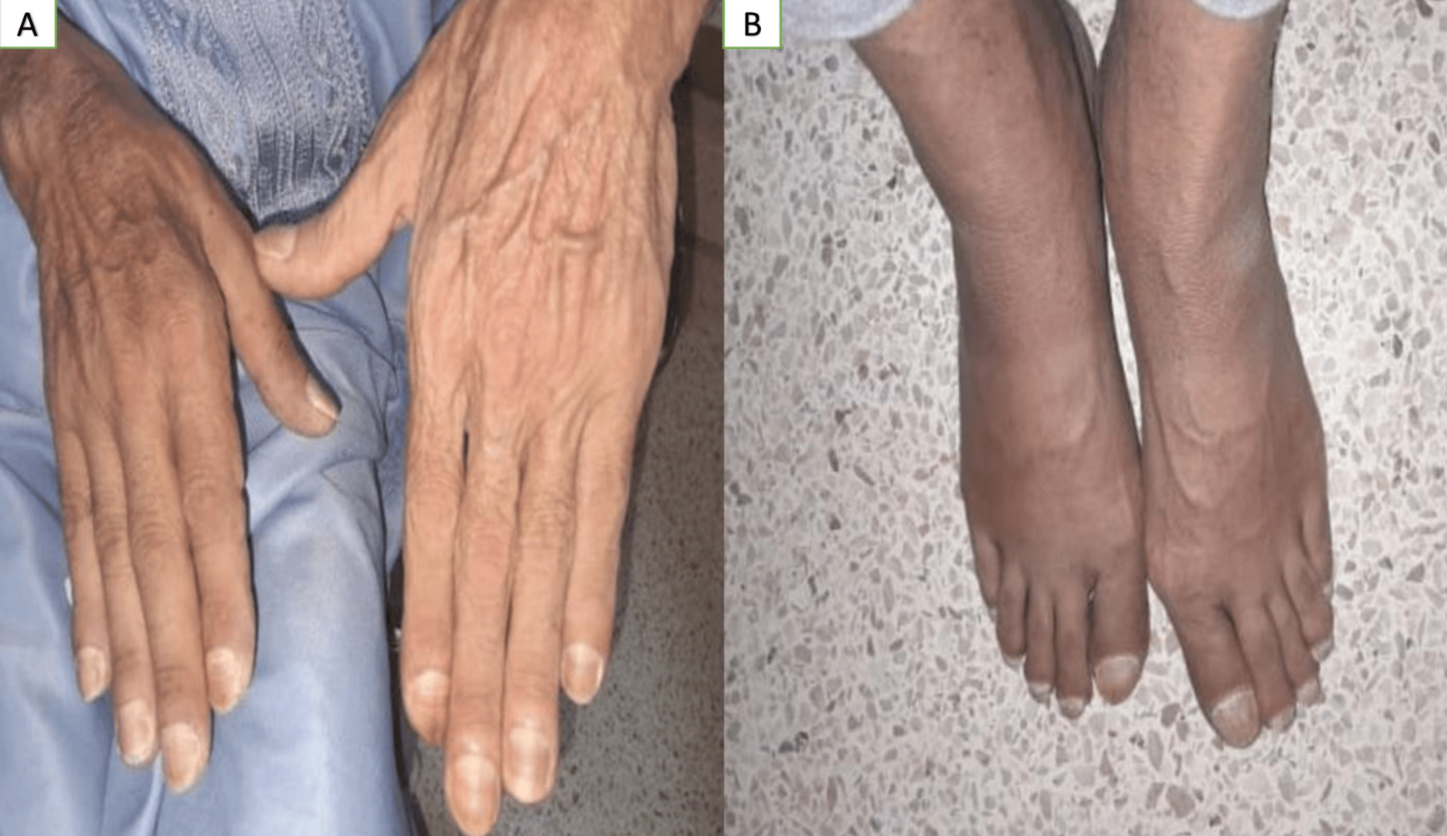
Pierre Marie-Bamberger syndrome's clinical osteoarticular manifestations A photograph of the patient’s hands and feet showing (A) digital clubbing and (B) pachydermia of the feet.

A 54-year-old man who had previously used tobacco heavily (35 pack-years) but had stopped three months ago and whose hypertension is controlled with 5 mg of amlodipine daily was seen for two months because of excruciating articular inflammation and edema in all four extremities. Digital hippocratism and foot pachydermia were the most noticeable clinical symptoms (Figure 1).

An additional anamnesis showed a progressive weight loss of 10 kg over three months, along with complaints of exertional dyspnea and dry cough that caused left-sided chest pain, without a family history of cancer or inflammatory rheumatism. Clinical examination of the respiratory system was within the norm.

The results of laboratory tests showed moderate inflammation with a C-reactive protein of 34 mg/L, and tests of liver function were within normal limits (Table 1).

**Table 1:** Summary of key laboratory findings with corresponding reference ranges and interpretations

Parameter	Patient value	Reference range	Interpretation
Hemoglobin	12.1 g/dL	12-16 g/dL	Normal
White blood cell count	9700/mm³	4,000-11,000/mm³	Normal
Platelet count	284000/mm³	150,000-400,000/mm³	Normal
C-reactive protein	34 mg/L	<5 mg/L	Moderate elevation
Urea	0.26 g/L	0.15-0.4 g/L	Normal
Creatinine	7.63 mg/L	5-9 mg/L	Normal
Aspartate aminotransferase	20 IU/L	<40 IU/L	Normal
Alanine aminotransferase	18 IU/L	<40 IU/L	Normal
Total bilirubin	2.9 mg/L	<10	Normal
Alkaline phosphatase	93 IU/L	30-100 IU/L	Normal

**Figure 2:**
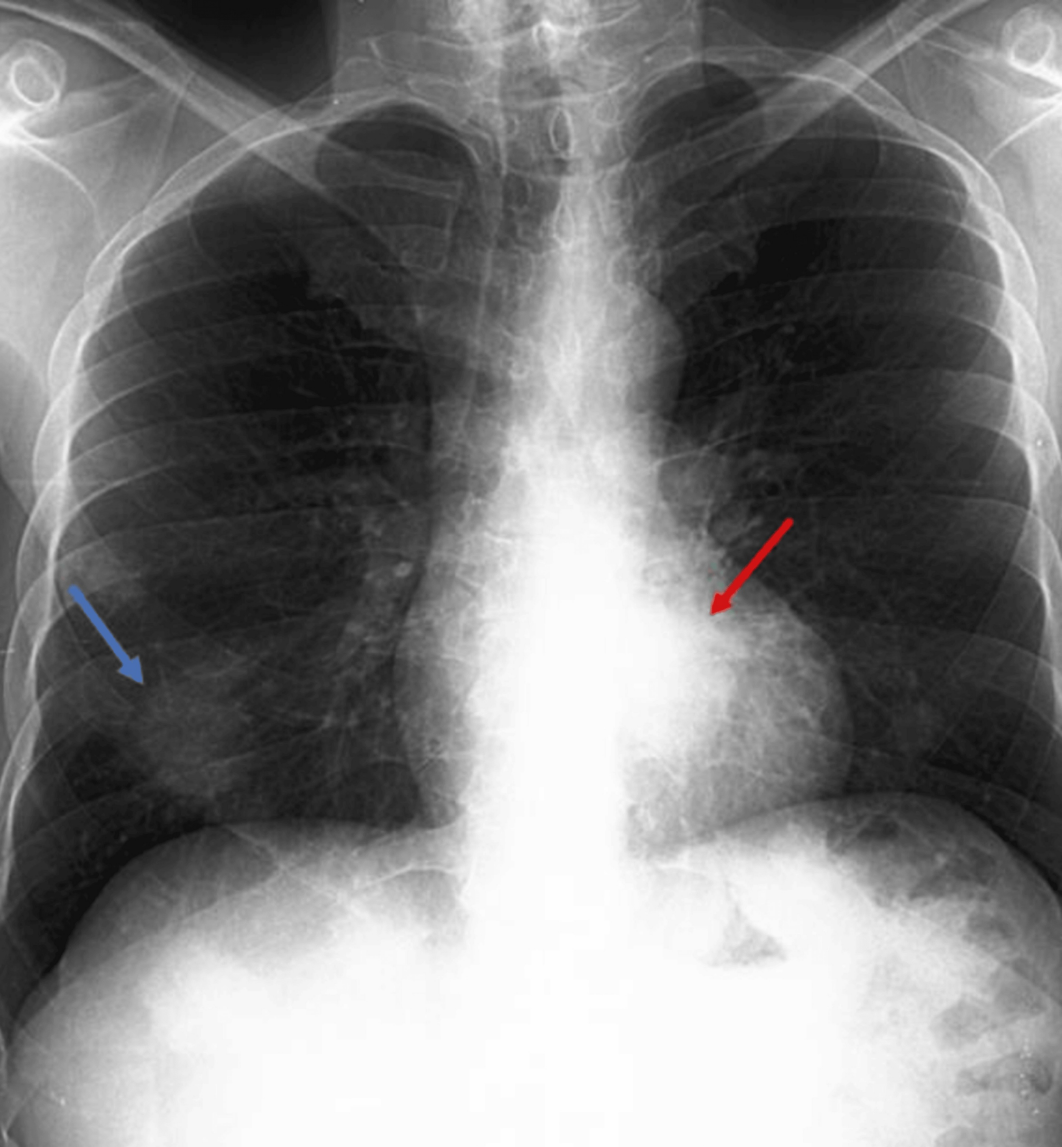
Chest X-ray The blue arrow indicates the right lower zone in a supradiaphragmatic position, which had the largest parenchymal opacity and measured 40 mm. The red arrow indicates a distinct oval opacity with sharp external borders and blurry internal margins that blended into the mediastinum, which was seen in the left lung base and retrocardiac on the same side.

Three parenchymal opacities were seen on the chest X-ray, in the middle and lower right lung fields. The largest measured 40 mm and was situated in a retrohepatic position. Furthermore, a distinct oval opacity with sharp external borders and blurry internal margins that blended into the mediastinum was seen in the left lung base and retrocardiac on the same side. The silhouette of the cardiomediastinal region was normal (Figure 2).

**Figure 3:**
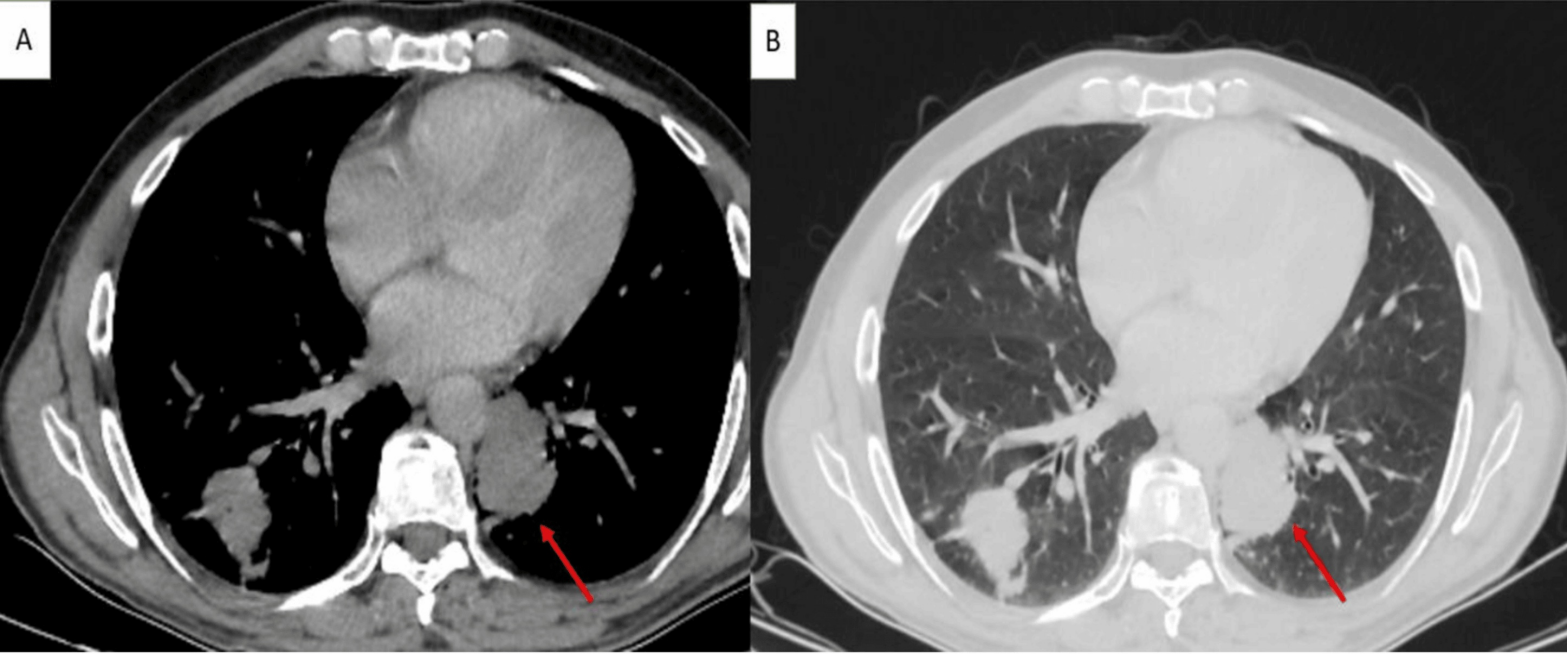
Thoracic CT scan In axial sections, displayed in mediastinal window (A) and lung window (B), after contrast injection, showing multiple bilateral and diffuse pulmonary parenchymal lesions. The largest lesion is located in the left costovertebral gutter, adjacent to the descending aorta, originating from the superior segment of the left lower lobe (red arrow), with soft tissue density and no significant enhancement after contrast administration.

Thoracic CT imaging revealed multiple tissue lesions, mostly necrotic, that primarily affected the lower lobes and involved both lung fields. The largest lesion, which measured 39 x 33 mm, was situated at the left costovertebral gutter, where it met the descending aorta at the expense of the superior segment of the left lower lobe (Figure 3).

The presence of a lung adenocarcinoma was confirmed by a CT-guided percutaneous biopsy of the right lesion

**Figure 4:**
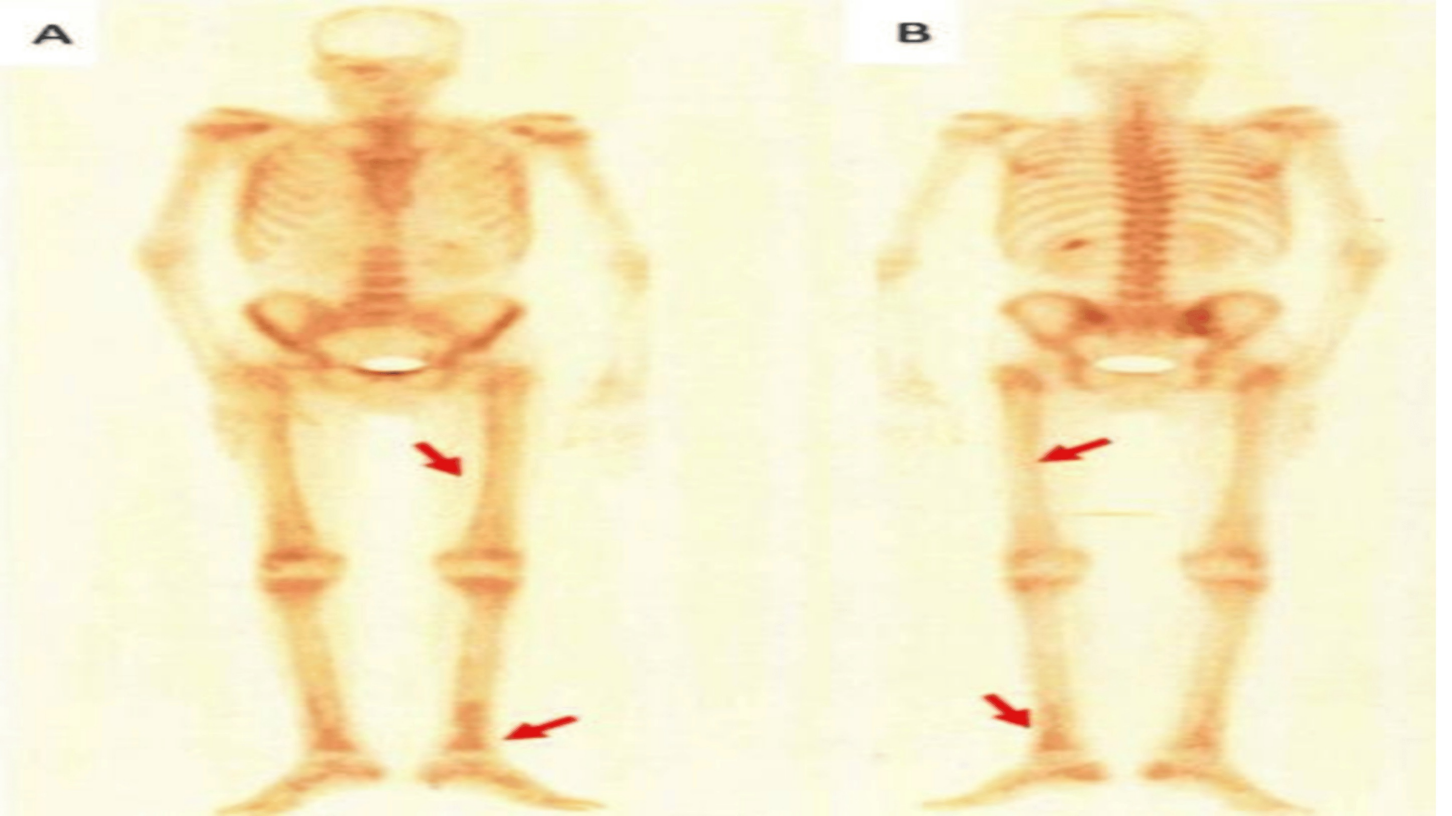
Bone scintigraphy: anterior (A) and posterior (B) faces Late images of the whole body show increased uptake in the axial and peripheral skeleton, more pronounced at the cortical regions of the lower limbs (red arrow).

PMB syndrome was strongly suggested by bone scintigraphy, which revealed increased uptake in the lower limbs' cortical bone, with a higher concentration in the axial and peripheral skeleton (Figure 4).

Following discussion during our institution’s multidisciplinary oncological board, palliative chemotherapy was administered to the patient, who showed signs of initial edema regression.

## Discussion

After being first described by Bamberger in 1889 and then further described by Pierre Marie in 1890, HOA was dubbed PMB disease [1-2]. Hypertrophy of bone and periarticular soft tissues is a hallmark of this rheumatologic condition, which is mostly caused by subperiosteal new bone formation, especially at the distal ends of long bones [4]. Digital clubbing, limb enlargement, arthralgia with edema, bilateral eyelid ptosis, pachydermia, and occasionally leonine facies are the clinical manifestations of the condition. Skin thickening and sebaceous hyperplasia are examples of cutaneous features, whereas subcutaneous tissue hyperplasia is usually revealed by histopathological analysis [5].

There are, in fact, two distinct kinds of HOA: primary and secondary. The primary form is a rare autosomal dominant hereditary disorder that is also known as pachydermoperiostosis [6]. Many conditions can cause secondary HOA, but the most common ones are paraneoplastic syndromes linked to primary or metastatic lung cancer. Nearly 80% of secondary HOA cases are caused by lung adenocarcinoma [7]. Our patient was 54 years old, but the condition has no discernible gender preference and typically manifests between the ages of 55 and 75 [8]. Secondary HOA typically develops more quickly and is more often painful than the primary form [4].

Infections, systemic diseases, and extra-pulmonary conditions like cardiovascular disease, cirrhosis, and chronic inflammatory bowel disease have all been mentioned as non-neoplastic lung diseases [9].

Circulating growth factors are believed to play a key role in secondary HOA by activating vascular and neurogenic pathways, despite the fact that the pathophysiology of this condition is not entirely understood. Two primary mechanisms have been proposed for the vascular pathway, specifically in HOA linked to lung cancer: (1) tumor overproduction of vasoactive mediators or hypoxemia and (2) systemic dissemination of these substances through pulmonary arteriovenous shunts. Vascular endothelial growth factor (VEGF), platelet-derived growth factor (PDGF), prostaglandin E2 (PGE2), growth hormone, estrogen, and adrenocorticotropic hormone are among the growth factors that have been linked. These mediators stimulate angiogenesis, vasodilation, fibroblast proliferation, endothelial cell activation, interstitial edema, and collagen deposition in periosteal and soft tissues [10-12].

Because it can identify abnormalities earlier and more sensitively than traditional radiography, bone scintigraphy is regarded as the preferred diagnostic modality [13]. The hallmark finding is a bilateral, symmetrical, linear tracer uptake along the cortical margins of long bones, which is commonly known as the "tram-line" or "double-stripe" sign [14]. It usually indicates periosteal involvement.

PMB syndrome has been reported to improve or resolve after the underlying disease has been cured, whether by definitive chemotherapy, radiotherapy, or surgical resection. On the other hand, symptoms usually continue and frequently worsen in patients who are only receiving palliative care and not the primary cause is being treated [15].

## Conclusions

Both clinical evaluation and radiographic evidence are necessary for the diagnosis of HOA. It should be regarded as a paraneoplastic manifestation until it is ruled out because it is most frequently linked to lung cancer. Early detection of HOA improves prognosis and directs appropriate therapeutic management by triggering the investigation for underlying pulmonary malignancy. To prevent delayed diagnosis, clinician awareness is essential. Timely interventions can be facilitated by identifying the distinctive clinical and radiographic signs. Additional research could enhance patient outcomes and shed light on the pathophysiology.

## References

[1] von Bamberger E (1889). Changes in the long bones in bronchiectasis [Article in French]. Wien Klin Wochenschr.

[2] Marie P (1890). On hypertrophic pulmonary osteoarthropathy [Article in French]. Rev Med Paris.

[3] Abbas S, Zil-E-Ali A, Ahmad U, Anwar K (2020). Pierre Marie-Bamberger syndrome: a unique case report. Liaquat Med Res J.

[4] Cannavò SP, Guarneri C, Borgia F, Vaccaro M (2005). Pierre Marie-Bamberger syndrome (secondary hypertrophic osteoarthropathy). Int J Dermatol.

[5] Fjouji S, Bakkali H, Houba A, Kartit N, Doghmi N, Balkhi H (2024). Pierre Marie-Bamberger or hypertrophic osteoarthropathy syndrome: paraneoplastic syndrome revealing lung tumor. J Clin Images Med Case Rep.

[6] Tan I, Lomasney L, Stacy GS, Lazarus M, Mar WA (2019). Spectrum of voriconazole-induced periostitis with review of the differential diagnosis. AJR Am J Roentgenol.

[7] Condé K, Guelngar C, Mohamed A (2021). Secondary hypertrophic osteoarthropathy or Pierre-Marie syndrome Bamberger: clinical case and literature review. Open J Rheumatol Autoimmune Dis.

[8] Ito T, Goto K, Yoh K (2010). Hypertrophic pulmonary osteoarthropathy as a paraneoplastic manifestation of lung cancer. J Thorac Oncol.

[9] Meyer HJ, Leifels L, Bach AG, Surov A (2017). Secondary hypertrophic osteoarthropathy caused by non-pleural or pulmonary tumors. Medicine (Baltimore).

[10] Davis MC, Sherry V (2011). Hypertrophic osteoarthropathy as a clinical manifestation of lung cancer. Clin J Oncol Nurs.

[11] Chakraborty RK, Sharma S (2018). Secondary hypertrophic osteoarthropathy. StatPearls [Internet].

[12] Kozak KR, Milne GL, Bentzen SM, Yock TI (2012). Elevation of prostaglandin E2 in lung cancer patients with digital clubbing. J Thorac Oncol.

[13] Openshaw MR, Rowan CS, Grumett S (2013). Atypical hypertrophic osteoarthropathy as a presenting complaint in a non-smoker. J Med Cases.

[14] Kumari P, Yeung P, Medani A, Kiani AN (2018). Hypertrophic pulmonary osteoarthropathy: an unusual presentation. Rheumatol Adv Pract.

[15] Koliakos E, Chappalley D, Kalogiannis E, Sgardello S, Christodoulou M (2023). Pierre-Marie Bamberger syndrome leading to the diagnosis and surgical treatment of a localized lung cancer. Cureus.

